# When Missing Out Matters: Associations Between Social Activities and Caregiver Burden in National Data

**DOI:** 10.1093/geroni/igaf122.731

**Published:** 2025-12-31

**Authors:** Mara Rosenberg, Alexander Smith, Ashwin Kotwal

**Affiliations:** University of California, San Francisco, San Francisco, California, United States; University of California, San Francisco, San Francisco, California, United States; University of California, San Francisco, San Francisco, California, United States

## Abstract

Engaging in social activities is essential for well-being, yet caregivers often face restrictions due to caregiving responsibilities. We analyzed cross-sectional data from the National Study of Caregiving Round 11 (N = 1,619, weighted N = 17.9 million) to examine the association between social restriction and burden among unpaid caregivers of adults aged 65+. Caregivers reported social activity participation (visiting friends/family, attending religious services, going out, group activities), the importance of these, and burden (emotional, physical, financial). Using weighted logistic regressions adjusting for sociodemographic covariates, we examined associations between restriction and burden. A subgroup analysis compared missing important versus unimportant activities. Average age was 65 years, 65% were female, 53% children of their care partner, 19% spouses. 43% reported any burden: 35% emotional, 17% physical, 9% financial. Caregivers kept from activities reported higher burden: when kept from going out (Emotional: 76% vs 43%; Physical: 37% v 16%; Financial: 24% v 7%), visiting family/friends (Emotional: 79% vs 38%; Physical: 41% v 14%; Financial: 15% v 6%), or group activities (Emotional: 82% vs 42%; Physical: 53% v 14%; Financial: 15% v 7%). The more missed activities, the greater any burden (37% v. 68% v. 81% for missing zero, one, or two, p < 0.05). Among religious activities, burden was higher if caregivers valued the missed activity (Emotional: 91% v. 47%; Physical: 64% v. 22%, p < 0.05). Findings underscore the need for personalized recommendations that align with caregiver preferences, while recognizing that any social interaction is beneficial. Providers should screen for burden when caregivers restrict participation and offer supportive resources.

